# Altered Expression of Brain-specific Autism-Associated miRNAs in the Han Chinese Population

**DOI:** 10.3389/fgene.2022.865881

**Published:** 2022-03-07

**Authors:** Ziqi Wang, Tianlan Lu, Xianjing Li, Miaomiao Jiang, Meixiang Jia, Jing Liu, Dai Zhang, Jun Li, Lifang Wang

**Affiliations:** ^1^ National Clinical Research Center for Mental Disorders (Peking University Sixth Hospital), NHC Key Laboratory of Mental Health (Peking University), Peking University Sixth Hospital, Peking University Institute of Mental Health, Beijing, China; ^2^ Chinese Institute for Brain Research, Beijing, China; ^3^ Guangdong Key Laboratory of Mental Health and Cognitive Science, Institute for Brain Research and Rehabilitation (IBRR), South China Normal University, Guangzhou, China

**Keywords:** autism, miRNAs, miRNA expression profiling, qRT-PCR, plasma

## Abstract

Autism is a complex neurodevelopmental disorder. However, its etiology is still unknown. MicroRNAs (miRNAs) are key post-transcriptional regulators. They play an important role in neurodevelopment and brain functions and may be involved in the pathogenesis of autism. Previous studies indicated altered expression of miRNAs in patients with autism. However, the findings were not consistent, and further explorations were needed. This study aimed to investigate whether miRNAs were dysregulated in autism. We examined the expression of 30 brain-specific autism-associated miRNAs in 110 patients with autism and 113 controls in the Han Chinese population using quantitative reverse transcription–polymerase chain reaction. The results demonstrated that 10 miRNAs (hsa-miR-191-5p, hsa-miR-151a-3p, hsa-miR-139-5p, hsa-miR-181a-5p, hsa-miR-432-5p, hsa-miR-181b-5p, hsa-miR-195-5p, hsa-miR-328-3p, hsa-miR-106a-5p, and hsa-miR-484) were significantly differentially expressed (false discovery rate <0.05). All of them were up-regulated in patients with autism compared with controls. The targets of these miRNAs were enriched for genes and pathways related to neurodevelopment, brain functions and autism. These findings suggested the participation of these 10 miRNAs in the pathogenesis of autism in the Han Chinese population.

## Introduction

Autism spectrum disorder (ASD) is a group of neurodevelopmental disorders. The core symptoms of ASD include significant deficits in social communication and interaction, repetitive behaviors, and restricted interests that commonly appear within the first 3 years of life and last throughout life. Autism affects about 1–2% of the world population with a higher incidence in boys than in girls (Maenner et al., 2020). In China, the prevalence of autism was estimated as 0.7% among 6- to 12-year-old children (Zhou et al., 2020). Studies have shown heritability of 80–90% in ASD; hundreds of genes and loci were indicated to be associated with the disorder (Geschwind, 2011). Recently, noncoding RNAs (ncRNAs), particularly microRNAs (miRNAs), were implicated in the central nervous system (CNS) functions and likely influenced the development of autism (Fabian and Sonenberg, 2012; Treiber et al., 2019). However, the contributions of these ncRNAs in autism are not completely understood yet.

MiRNAs comprise a class of evolutionarily conserved ncRNAs consisting of 19–25 nucleotides. They play an important role in post-transcriptional gene silencing and participate in a multitude of biological processes through base pairing to the 3′ untranslated regions of target messenger RNAs (mRNAs) to degrade the mRNAs or inhibit the translation (Fabian and Sonenberg, 2012; Treiber et al., 2019). A single miRNA could bind to multiple mRNAs. Together, these miRNAs could regulate around two thirds of human mRNAs, and 70% of them were expressed in the CNS (Tonacci et al., 2019). MiRNAs might be essential regulators in brain functions including neuronal plasticity and neuronal development (Rajasethupathy et al., 2009; Follert et al., 2014). Previous studies suggested the association between a number of miRNAs and various neuropsychological diseases including autism (Kocerha et al., 2015; Van Den Berg et al., 2020). Significant changes were detected in the expression of miRNAs in patients with autism using a variety of biomaterials such as postmortem brain, peripheral blood, and saliva. These dysregulated miRNAs might affect the expression of genes related to autism and neurodevelopment (Sarachana et al., 2010; Mor et al., 2015; Wu et al., 2016).

However, most of the current studies about the expression changes of miRNAs in autism focused on European and American individuals. Only two studies explored the miRNA expression in Chinese individuals, both using peripheral blood samples with a relatively small sample size. However, the findings of these studies were not consistent. To explore whether these potentially autism-associated miRNAs were dysregulated in autism, we selected brain-specific miRNAs with at least two consistent reports and analyzed their expression profiles in patients with autism and healthy controls in the Han Chinese population.

## Materials and Methods

### Participants

This study included 110 patients with autism (93 male and 17 female) and 113 typically developing unrelated age- and sex-matched controls (95 male and 18 female). All participants were of Han Chinese ancestry and recruited at the Peking University Sixth Hospital (Beijing, China). The median age of diagnosis for children with autism was 4.39 (range 3.07–5.97) years.

Only children with typical autism were recruited to decrease heterogeneity. They should meet the following criteria under the independent assessment by two senior child psychiatrists: 1) fulfilling the Diagnostic and Statistical Manual of Mental Disorders, fourth edition criteria for autism; 2) Autism Behavior Checklist score ≥53; and 3) Childhood Autism Rating Scale score ≥35 (Krug et al., 1980; Schopler et al., 1980). Healthy controls were evaluated by two psychiatrists through unstructured interviews to confirm that they were not affected by autism. Any participant with Asperger syndrome, pervasive developmental disorder not otherwise specified, fragile X syndrome, tuberous sclerosis, a previously identified chromosomal abnormality, other neurological conditions, familial/inherited diseases (such as congenital deaf-mutism, hemophilia, and familial adenomatous polyposis), or severe mental disorders (such as schizophrenia, schizoaffective disorder and bipolar disorder) was excluded from the present study.

### RNA Extraction and Quantitative Reverse Transcription–Polymerase Chain Reactions

The peripheral blood samples were collected from all participants and then centrifuged at 3,500 rpm at 4°C for 10 min to separate plasma from blood cells. Total RNA was extracted from 200-μL plasma samples using the Qiagen miRNeasy Serum/Plasma Kit (Qiagen, GmbH, Hilden, Germany) following the manufacturer’s protocols. The *Caenorhabditis elegans* miR-39 (cel-miR-39) mimic from miRNeasy Serum/Plasma Spike-in Control (Qiagen) was added to the lysed samples for internal normalization. The total RNA samples were then reverse transcribed with TransGen TransScript miRNA First-Strand cDNA Synthesis SuperMIX (TransGen, Beijing, China). Each cDNA was further diluted with RNase-free water and stored at −20°C until use. Quantitative reverse transcription–polymerase chain reaction (qRT-PCR) was performed with TransGen PerfectStar Green qPCR SuperMix (TransGen, Beijing, China) on a LightCycler 96 Instrument (Roche, Switzerland). The qRT-PCR was performed in triplicate with a preincubation of 94°C for 30 s, followed by 45 cycles of 94°C for 5 s and 60°C for 30 s. Data were normalized where appropriate with the exogenous control cel-miR-39. All primers are listed in Supplementary Table S1. The relative quantitation for miRNA was calculated using the 2^−∆∆Ct^ method.

### Autism-Associated and Brain-specific miRNA Selection

PubMed, Google Scholar, and Web of Science databases were searched for case−control studies exploring the differentially expressed miRNAs between patients with autism and healthy controls using the search terms of “microRNA/miRNA” AND “autism/autism spectrum disorder” until 1 November 2021. The significant threshold values of *p* and fold change (FC) were set according to the original studies.

Human miRNA expression data were obtained from the miRmine database (http://guanlab.ccmb.med.umich.edu/mirmine). It comprised miRNA expression data from 304 high-quality experiments, including 16 different types of human tissues and biofluids: bladder, blood, brain, breast, hair follicle, liver, lung, nasopharynx, pancreas, placenta, plasma, saliva, semen, serum, sperm and testis (Panwar et al., 2017). Brain-specific miRNAs were defined as miRNAs whose expression in the brain ranked in the top five among all tissues and were higher than that of 90% of all miRNAs in the brain (Teng et al., 2020).

### Bioinformatics Analysis

The targets of each miRNA were predicted with MiRWalk 3.0 (http://mirwalk.umm.uni-heidelberg.de/). The genes were selected for subsequent analyses if the miRNA-mRNA interactions were experimentally validated or predicted using both TargetScan and miRDB.

Gene Ontology (GO) and KEGG pathway enrichment analyses for the target genes were performed using the R package clusterProfiler (Yu et al., 2012). ASD-related genes were obtained from the Human Gene Module of Simons Foundation Autism Research Initiative database (ASD_SFARI, https://gene.sfari.org/database/human-gene/), which comprised 1023 candidate ASD risk genes (2 September 2021, release). The genes affected by likely gene-disrupting (including nonsense, splice site, and frame-shift) and missense rare *de novo* variants (DNVs) detected in ASD were also included (ASD_DNVs_LGD, 353 genes; ASD_DNVs_missense, 1771 genes) (Iossifov et al., 2014). Further, 401 genes involved in the intellectual disability (ID_all) were acquired from the study by Parikshak, N. N. et al., 2013 (Parikshak et al., 2013). The genes in ASD_SFARI but not in ID_all (ASD_only), genes in both ASD_SFARI and ID_all (ASD&ID overlap), and genes in ID_all but not in ASD_SFARI (ID_only) were analyzed for gene sets enrichment. ASD-associated mRNA coexpression modules were indicated by Parikshak, N. N. et al., 2013, including 2 down-regulated modules ASD_CoexDown_M2 (1042 genes) and ASD_CoexDown_M3 (996 genes) and 3 up-regulated modules ASD_CoexDown_M13 (870 genes), ASD_CoexDown_M16 (492 genes) and ASD_CoexDown_M17 (1042 genes). The markers for different types of neural cells (neurons, 1484 genes; astrocytes, 1960 genes; oligodendrocytes, 1614 genes; microglias, 364 genes) were obtained from the study by Werling et al., 2016 (Werling et al., 2016). The fisher’s exact test was performed to evaluate whether a gene set was enriched over background (∼20,000 protein-coding genes in the whole genome). *p*-values were adjusted for multiple comparisons using Benjamini–Hochberg correction to assess the false discovery rate (FDR).

### Statistical Analyses

Data were analyzed using SPSS Statistics 24 and R 4.0.5. software. The homogeneity for age and sex between patients with autism and controls was assessed with the Student’s *t*-test and the chi-squared test following the examination of the normality of distribution using the Kolmogorov–Smirnov test. The differences in relative expression for each miRNA between patients with autism and controls were examined using nonparametric Mann–Whitney U test (two-tailed). FDR was used for multiple comparison corrections. The threshold of significance accepted for all statistical analyses was the *p*-value or FDR less than 0.05.

## Results

**FIGURE 1:**
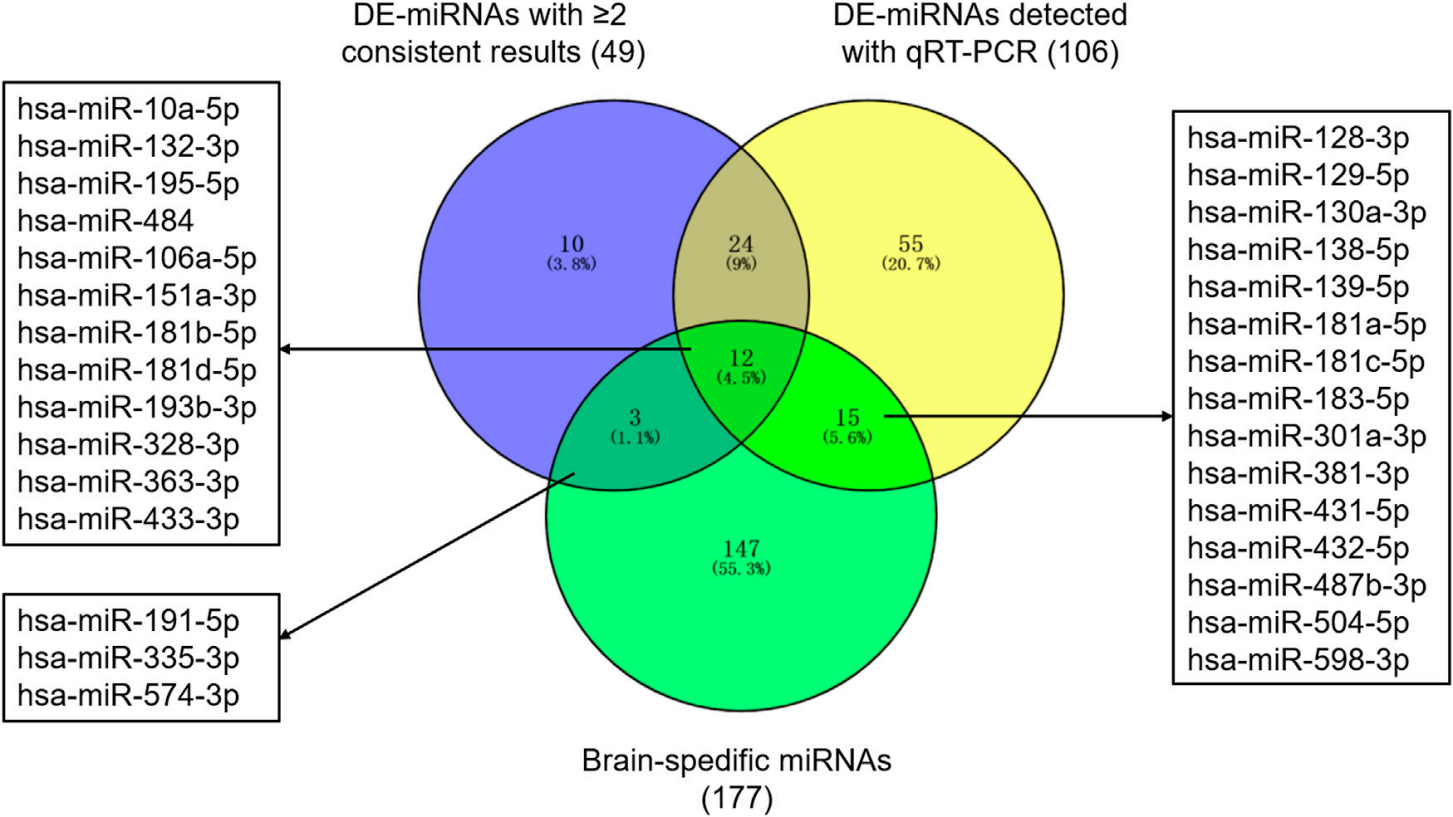
Autism-associated and brain-specific miRNAs. Abbreviations: DE-miRNAs, differentially expressed miRNAs; qRT-PCR, quantitative reverse–transcription polymerase chain reactions.

Based on previous studies, 311 miRNAs (DE-miRNAs) were indicated differential expression between patients with ASD and controls (Supplementary Table S2). 74 DE-miRNAs were described in multiple studies, of which 49 showed consistent up-regulation or down-regulation. Besides, 106 DE-miRNAs were detected with qRT-PCR. Together, 119 miRNAs were selected as autism-associated miRNAs (Figure 1). Using transcription data from the miRmine database, we defined 177 miRNAs as brain-specific miRNAs (Supplementary Table S3). We selected 30 miRNAs that were both autism-associated and brain-specific for further validation (Figure 1 and Supplementary Table S4).

**FIGURE 2:**
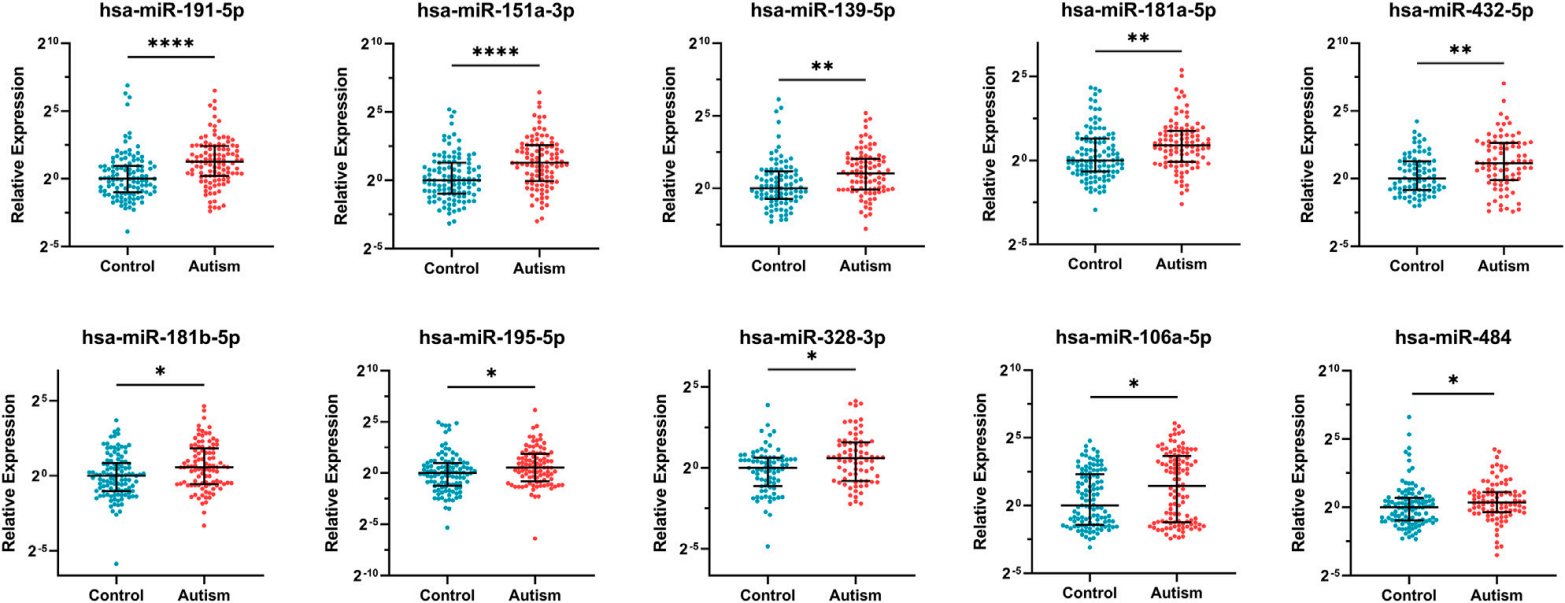
Expression of 10 significantly differentially expressed miRNAs in patients with autism and healthy controls. Mann–Whitney U test was used for statistical analysis and false discovery rate (FDR) was used for multiple testing correction. Data are presented as the median and interquartile range (lines) with each dot representing an individual. * FDR <0.05, ** FDR <0.01, **** FDR <0.0001.

10 DE-miRNAs exhibited significantly differential expression between patients with autism and controls (FDR <0.05). All of them were up-regulated. The expression of five miRNAs in autism increased to greater than twofold of that in the controls, including hsa-miR-191-5p (FC = 2.40, FDR = 2.63E-05), hsa-miR-151a-3p (FC = 2.45, FDR = 4.67E-05), hsa-miR-139-5p (FC = 2.03, FDR = 2.06E-03), hsa-miR-432-5p (FC = 2.22, FDR = 4.60E-03), and hsa-miR-106a-5p (FC = 2.73, FDR = 0.03; Table 1 and Figure 2). Considering the difference in prevalence and clinical manifestations between male and female patients with autism, we further compared the expression of these miRNAs between patients with autism and controls using samples only from males. Six of the 10 DE-miRNAs were significantly dysregulated, including hsa-miR-191-5p, hsa-miR-151a-3p, hsa-miR-139-5p, hsa-miR-181a-5p, hsa-miR-432-5p, and hsa-miR-195-5p. All of them were increasingly expressed in patients with autism (Supplementary Table S5 and Supplementary Figure S1).

**TABLE 1:** Expression of 30 brain-specific autism-associated miRNAs in patients with autism and controls.

miRNA	FC	*p* ^a^	FDR^b^	Regulation	miRNA	FC	*p* ^a^	FDR^b^	Regulation
hsa-miR-191-5p	2.40	8.76E-07	**2.63E-05**	up	hsa-miR-335-3p	1.37	0.11	0.20	up
hsa-miR-151a-3p	2.45	3.11E-06	**4.67E-05**	up	hsa-miR-431-5p	1.56	0.13	0.21	up
hsa-miR-139-5p	2.03	2.06E-04	**2.06E-03**	up	hsa-miR-128-3p	2.55	0.14	0.21	up
hsa-miR-181a-5p	1.85	4.30E-04	**3.23E-03**	up	hsa-miR-130a-3p	1.34	0.14	0.21	up
hsa-miR-432-5p	2.22	7.67E-04	**4.60E-03**	up	hsa-miR-181d-5p	1.39	0.14	0.21	up
hsa-miR-181b-5p	1.48	3.47E-03	**0.02**	up	hsa-miR-183-5p	1.61	0.16	0.22	up
hsa-miR-195-5p	1.45	8.18E-03	**0.03**	up	hsa-miR-129-5p	1.26	0.17	0.24	up
hsa-miR-328-3p	1.51	9.37E-03	**0.03**	up	hsa-miR-598-3p	1.54	0.23	0.30	up
hsa-miR-106a-5p	2.73	9.93E-03	**0.03**	up	hsa-miR-574-3p	2.03	0.28	0.33	up
hsa-miR-484	1.28	1.09E-02	**0.03**	up	hsa-miR-138-5p	0.73	0.28	0.33	down
hsa-miR-363-3p	1.37	2.71E-02	0.07	up	hsa-miR-181c-5p	1.48	0.33	0.38	up
hsa-miR-193b-3p	0.40	2.80E-02	0.07	down	hsa-miR-381-3p	0.44	0.40	0.44	down
hsa-miR-504-5p	0.63	3.15E-02	0.07	down	hsa-miR-10a-5p	1.06	0.49	0.52	up
hsa-miR-132-3p	1.47	3.62E-02	0.08	up	hsa-miR-487b-3p	0.98	0.76	0.78	down
hsa-miR-433-3p	1.56	4.58E-02	0.09	up	hsa-miR-301a-3p	1.06	0.88	0.88	up

Abbreviations: FC, fold change; FDR, false discovery rate.

a Mann–Whitney U test (two-tailed).

b Bold values indicate FDR <0.05.

**FIGURE 3:**
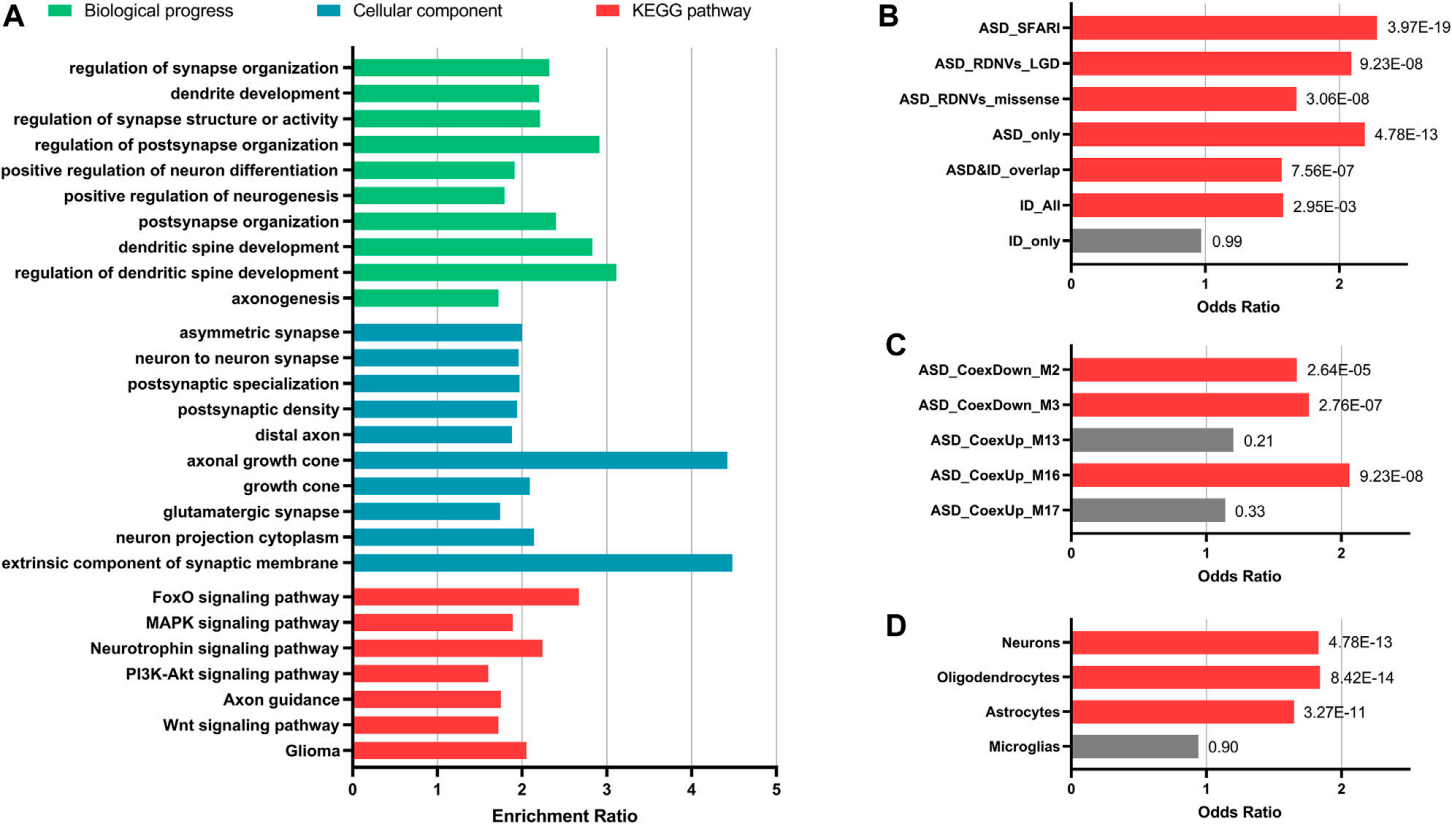
Enrichment analyses for the target genes of 10 significantly differentially expressed miRNAs. **(A)** Enrichment for GO and KEGG pathways that were involved in brain functions. **(B–D)** Enrichment for ASD-related gene lists **(B)**, ASD-associated gene coexpression modules **(C)**, and markers for different types of neural cells **(D)**. Fisher’s exact test (two-tailed) with false discovery rate (FDR) correction was applied for enrichment. FDR value of each gene set was indicated. Gene sets with FDR <0.05 are showed with red bars and the others are showed with grey bars. Abbreviations: ASD, autism spectrum disorder; ID, intelligent disability; DNVs, *de novo* variants; LGD, likely gene-disrupting.

The targets prediction revealed miRNA–mRNA interactions between the 10 significant DE-miRNAs and 1732 genes. The GO analysis indicated that these target genes were significantly related to neurogenesis, neuron projection, and synapse functions (FDR <0.05). For KEGG pathway analysis, the target genes over-represented multiple brain-related KEGG pathways including MAPK signaling pathway (FDR = 2.87E-04), PI3K–Akt signaling pathway (FDR = 4.46E-03), axon guidance (FDR = 0.01), and Wnt signaling pathway (FDR = 0.02, Figure 3A). The 10 miRNAs also targeted 176 candidate ASD risk genes from the SFARI database [FDR = 3.97E-19, OR (95% CI) = 2.28 (1.93–2.69)] and genes harboring rare DNVs detected in ASD, including representative ASD-related genes such as *MECP2* (hsa-miR-106a-5p, hsa-miR-181a-5p, hsa-miR-195-5p, and hsa-miR-328-3p), *FMR1* (hsa-miR-139-5p, hsa-miR-181a-5p, and hsa-miR-181b-5p), *DDX3X* (hsa-miR-139-5p and hsa-miR-181b-5p), *PTEN* (hsa-miR-181a-5p and hsa-miR-181b-5p), and *RELN* (hsa-miR-195-5p) (Figure 3B and Supplementary Table S6). Besides, the target genes were significantly enriched for ASD-associated mRNA coexpression down-regulated modules ASD_CoexDown_M2 and ASD_CoexDown_M3 and up-regulated module ASD_CoexUp_M16, as well as markers for neurons, oligodendrocytes, and astrocytes (FDR <0.05, Figures 3C,D).

## Discussion

This study investigated the altered expression of 30 brain-specific ASD-associated miRNAs in a Han Chinese cohort. The results revealed that 10 DE-miRNAs (hsa-miR-191-5p, hsa-miR-151a-3p, hsa-miR-139-5p, hsa-miR-181a-5p, hsa-miR-432-5p, hsa-miR-181b-5p, hsa-miR-195-5p, hsa-miR-328-3p, hsa-miR-106a-5p, and hsa-miR-484) were significantly differentially expressed in plasma of patients with autism than in controls (FDR <0.05). All the 10 DE-miRNAs were up-regulated in autism and might be involved in neurodevelopment and multiple brain-related functions and pathways. Besides, the targets of these miRNAs were significantly enriched in ASD-related genes.

Previous studies proved that circulating miRNAs ubiquitously existed in different body fluids, including the peripheral blood plasma (Weber et al., 2010). Plasma or brain miRNAs could physically cross the blood–brain barrier (BBB), and the BBB endothelium also released miRNAs into the circulation (Langford and Masliah, 2001; Witwer et al., 2011). Circulating miRNAs might reflect the pathogenesis of the brain. In the present study, 10 miRNAs were found to be significantly differentially expressed (FDR <0.05), suggesting the altered expression levels of circulating miRNAs in autism. Five of them (hsa-miR-191-5p, hsa-miR-181a-5p, hsa-miR-181b-5p, hsa-miR-195-5p, and hsa-miR-328-3p) were detected consistent up-regulation in the peripheral blood of patients with autism in the previous studies. Besides, the up-regulation of hsa-miR-484 was described in two studies using postmortem brain samples (Supplementary Table S4). These findings suggested the potential of circulating miRNAs to be biomarkers for the diagnosis of autism.

We further explored the role of 10 DE-miRNAs targets in brain-related functions and autism. The results showed that these DE-miRNAs might regulate genes and pathways involved in neurodevelopment and neuronal and synaptic functions. The DE-miRNAs targets were significantly enriched for ASD-related genes but not for ID-only genes, indicating a stronger association for ASD and a relatively weaker association for ID. As for ASD-associated gene coexpression modules, the targets of DE-miRNAs were enriched for two down-regulated modules (ASD_CoexDown_M2 and ASD_CoexDown_M3), which were most highly expressed in early human fetal development. Besides, these brain-specific DE-miRNAs might bind to the markers for neurons, oligodendrocytes, and astrocytes, but were not associated with microglia, suggesting that these up-regulated miRNAs were more involved in neuronal functions.

Previous studies demonstrated the regulatory roles of these DE-miRNAs in neurodevelopment and brain functions. For example, hsa-miR-139-5p might act as a negative regulator for neural stem cell proliferation and neuronal differentiation, and modulate cortical neuronal migration by targeting *Lis1* (Huang et al., 2014; Wei et al., 2020). Hsa-miR-484 played an essential role in neurocognition and regulated mitochondrial functions crucial for maintaining synaptic function (Allach El Khattabi et al., 2020; Wingo et al., 2020). Hsa-miR-151a-3p was implicated in SSRI responsiveness and possibly in the clinical response to antidepressant drugs *via* down-regulating CHL1 expression (Oved et al., 2017). Hsa-miR-195-5p was increasingly expressed and targeted BDNF in rats with the rapid onset of heavy alcohol use (Ehinger et al., 2021). The altered expression of these DE-miRNAs was also found in individuals with other comorbid psychiatric disorders sharing genetic overlap with autism, including major depressive disorder, attention-deficit/hyperactivity disorder, and Alzheimer’s disease (Mendes-Silva et al., 2016; Sánchez-Mora et al., 2019; Zadehbagheri et al., 2019). These findings, along with those of the present study, supported the relationship between miRNAs and autism, suggesting that up-regulated miRNAs might inhibit the expression of down-regulated genes related to neuronal and synaptic functions (Gupta et al., 2014; Wu et al., 2016). The circulating miRNAs might have the potential to become biomarkers for the diagnosis of autism and provide clues for understanding the pathogenesis of the disorder. Further research is needed to investigate the expression profile and regulatory roles of miRNAs in the CNS and neuropsychiatric disorders.

Some miRNAs did not exhibit consistent changes across different studies. The possible influencing factors might include the type of biomaterials, expression measurement, population/ethnicity, heterogeneity of ASD, and limitation of sample sizes. Besides, most postmortem brain samples used in previous studies were from adults. Although the results were more likely to reflect the pathogenesis in the CNS, the variability in the miRNA expression profile could not be ignored (Mariani et al., 2015; Prieto-Fernández et al., 2020).

This study had several limitations. First, the sample size was relatively small and the miRNAs examined were limited. Second, only patients with typical autism were included in the present study; expression changes of miRNAs in patients with mild symptoms were not investigated. Third, the targets of DE-miRNAs were predicted based on the online database. Further experiments should be performed to identify the actual targets for these miRNAs.

In summary, this study suggested significantly altered expression of 10 miRNAs (hsa-miR-191-5p, hsa-miR-151a-3p, hsa-miR-139-5p, hsa-miR-181a-5p, hsa-miR-432-5p, hsa-miR-181b-5p, hsa-miR-195-5p, hsa-miR-328-3p, hsa-miR-106a-5p, and hsa-miR-484) in patients with autism. These miRNAs might be involved in neurodevelopment and brain functions. Further studies are required to explore the dysregulation of miRNAs in autism and its underlying mechanisms.

## Data Availability

The original contributions presented in the study are included in the article/Supplementary Material, further inquiries can be directed to the corresponding authors.
