# Supplementary material for: Altered Expression of Brain-specific Autism-Associated miRNAs in the Han Chinese Population
Source: Front Genet. 2022 Mar 7;13:865881. doi: 10.3389/fgene.2022.865881 (PMC8942769; doi:10.3389/fgene.2022.865881)
Supplement: Supplementary file 1 [file DataSheet1.docx]

**Altered Expression of Brain-specific Autism-associated miRNAs in the Han Chinese Population**

**Supplementary Materials**

**Table S1.** Primers of 30 miRNAs for qRT-PCR.

**Table S2.** Information of studies on differentially expressed miRNAs in autism.

**Table S3.** Brain-specific expressed miRNAs.

**Table S4.** Expression changes of 30 selected miRNAs reported in previous studies.

**Table S5.** Expression of 30 brain-specific autism-associated miRNAs in male samples.

**Table S6.** Target genes of 10 significantly differentially expressed miRNAs for ASD candidate risk genes from SFARI database.

**Figure S1.** Expression of 6 significantly differentially expressed miRNAs in male patients with autism and healthy controls.

**Table S1.** Primers of 30 miRNAs for qRT-PCR.

| miRNA | forward primer (5'-3') | miRNA | forward primer (5'-3') |
| --- | --- | --- | --- |
| hsa-miR-106a-5p | AAAAGTGCTTACAGTGCAG | hsa-miR-193b-3p | AACTGGCCCTCAAAGTC |
| hsa-miR-10a-5p | TACCCTGTAGATCCGAATTTG | hsa-miR-195-5p | GTAGCAGCACAGAAATATTGG |
| hsa-miR-128-3p | TCACAGTGAACCGGTCT | hsa-miR-301a-3p | CAGCAGTGCAATAGTATTGT |
| hsa-miR-129-5p | GGCTTTTTGCGGTCTGG | hsa-miR-328-3p | GCTGGCCCTCTCTG |
| hsa-miR-130a-3p | GCAGCAGTGCAATGTTA | hsa-miR-335-3p | GCAGTTTTTCATTATTGCTCCT |
| hsa-miR-132-3p | ACAGTCTACAGCCATGGT | hsa-miR-363-3p | AATTGCACGGTATCCATCT |
| hsa-miR-138-5p | GCTGGTGTTGTGAATCAG | hsa-miR-381-3p | TATACAAGGGCACGCTCTCT |
| hsa-miR-139-5p | TACAGTGCACGTGTCTC | hsa-miR-431-5p | TGTCTTGCAGTCCGTCATGC |
| hsa-miR-151a-3p | CTAGACTGAAGCTCCTTGAG | hsa-miR-432-5p | TTGGAGTAGGTCATTGGGT |
| hsa-miR-181a-5p | CGCAACATTCAACGCTGTC | hsa-miR-433-3p | GGGCTCCTCGGTG |
| hsa-miR-181b-5p | AACATTCATTGCTGTCGGT | hsa-miR-484 | CAGTCAGGCTCAGTCC |
| hsa-miR-181c-5p | CATTCAACCTGTCGGTGA | hsa-miR-487b-3p | ATCGTACAGGGTCATCCA |
| hsa-miR-181d-5p | GAACATTCATTGTTGTCGGT | hsa-miR-504-5p | AGACCCTGGTCTGCACTC |
| hsa-miR-183-5p | TATGGCACTGGTAGAATTCAC | hsa-miR-574-3p | CTCATGCACACACCCA |
| hsa-miR-191-5p | CGGAATCCCAAAAGCAG | hsa-miR-598-3p | AGTACGTCATCGTTGTCATC |

**Table S2.** Information of studies on differentially expressed miRNAs in autism.

| No. | Publications | Biomaterial | Participants |
| --- | --- | --- | --- |
| 1 | Abu-Elneel, K. *et al*., 2008 | postmortem cerebellar cortex | 13 ASD, 13 controls |
| 2 | Talebizadeh, Z. *et al*., 2008 | lymphoblastoid cell lines (LCLs) | 6 ASD, 6 controls |
| 3 | Sarachana, T. *et al*., 2010 | LCLs | 14 ASD, 14 sibling controls (3 monozygotic twins) |
| 4 | Ghahramani Seno, M. M. *et al*., 2011 | LCLs | 20 ASD, 22 sibling controls |
| 5 | Popov, N. T. *et al*., 2012 | whole blood | 30 autism, 25 controls |
| 6 | Mundalil Vasu, M. *et al*., 2014 | serum | 55 ASD, 55 controls |
| 7 | Ander, B. P. *et al*., 2015 | postmortem superior temporal sulcus, primary auditory cortex | 10 ASD, 8 controls |
| 8 | Huang, F. *et al*., 2015 | peripheral blood | 5 ASD/5 control (microarray), 15ASD/15 control (qRT-PCR) |
| 9 | Mor, M. *et al*., 2015 | postmortem BA 10 | 12 autism, 12 controls |
| 10 | Hicks, S. D. *et al*., 2016 | saliva | 24 ASD, 21 controls |
| 11 | Nguyen, L. S. *et al*., 2016 | olfactory mucosal stem cells, skin fibroblasts or PBMCs | 8 ASD, 6 controls |
| 12 | Wu, Y. E. *et al*., 2016 | postmortem frontal cortex (BA 9), temporal cortex (BA41/42/22) | 28 ASD, 28 controls |
| 13 | Cirnigliaro, M. *et al*., 2017 | serum, saliva | 4 ASD, 5 TS, 4 TS+ASD, 3 controls (serum); 30 ASD, 24 TS, 25 TS+ASD, 25 controls (saliva) |
| 14 | Kichukova, T. M. *et al*., 2017 | serum | 30 ASD, 30 controls |
| 15 | Pagan, C. *et al*., *et al*., 2017 | postmortem pineal glands, plasma | 9 ASD, 22 controls (pineal glands); 54 ASD, 70control (plasma) |
| 16 | Schumann, C. M. *et al*., 2017 | postmortem superior temporal sulcus, primary auditory cortex | 10 ASD, 8 controls |
| 17 | Hicks, S. D. *et al*., 2018 | saliva | 188 ASD, 184 controls (training set); 50 ASD, 34 controls (test set) |
| 18 | Nguyen, L. S. *et al*. 2018 | postmortem temporal lobe (BA 21) | 5 ASD, 6 controls |
| 19 | Nt, P. *et al*., 2018 | serum | 30 ASD, 30 controls |
| 20 | Vaccaro, T. D. S. *et al*., 2018 | peripheral blood | 7 male ASD, 4 male controls |
| 21 | Yu, D. *et al*., 2018 | peripheral blood | 2 ASD, 3 controls (microarray); 18 ASD, 20 controls (qRT-PCR) |
| 22 | Jyonouchi, H. *et al*., 2019 | serum | 105 ASD, 35 controls |
| 23 | Moore, D. *et al*., 2019 | iPSC-derived neuronal stem cells | 3 ASD, 3 controls |
| 24 | Nakata, M. *et al*., 2019 | peripheral blood | 30 high-functioning ASD, 30 controls |
| 25 | Almehmadi, K. A. *et al*., 2020 | postmortem amygdala and dorsolateral prefrontal cortex regions (DLPFC) | 8 ASD, 8 controls (amygdala); 7 ASD, 7 controls (DLPFC) |
| 26 | Hicks, S. D. *et al*., 2020 | saliva | 187 ASD, 125 TD, 69 non-autism DD (training set); 37 ASD, 8 TD, 17 non-autism DD (test set) |
| 27 | Frye, R. E. *et al*. 2021 | LCLs | 10 ASD, 7 controls, 10 siblings |

Abbreviations: PBMCs, peripheral blood mononuclear cells; iPSC, induced pluripotent stem cells; ASD, autism spectrum disorder; TS, Tourette Syndrome; TD, typical development; DD, developmental delay; qRT-PCR, quantitative reverse-transcription polymerase chain reactions.

**Table S3.** Brain-specific expressed miRNAs.

| hsa-let-7b-3p | hsa-miR-135a-3p | hsa-miR-216a-5p | hsa-miR-409-3p | hsa-miR-517c-3p | hsa-miR-525-3p |
| --- | --- | --- | --- | --- | --- |
| hsa-let-7b-5p | hsa-miR-135a-5p | hsa-miR-216b-5p | hsa-miR-410-3p | hsa-miR-518a-3p | hsa-miR-525-5p |
| hsa-let-7c-5p | hsa-miR-135b-5p | hsa-miR-217 | hsa-miR-411-5p | hsa-miR-518b | hsa-miR-526b-5p |
| hsa-let-7d-3p | hsa-miR-136-3p | hsa-miR-218-5p | hsa-miR-421 | hsa-miR-518c-3p | hsa-miR-532-3p |
| hsa-let-7e-5p | hsa-miR-137 | hsa-miR-219a-1-3p | hsa-miR-423-3p | hsa-miR-518c-5p | hsa-miR-574-3p |
| hsa-miR-106a-5p | hsa-miR-138-5p | hsa-miR-219a-2-3p | hsa-miR-431-5p | hsa-miR-518d-3p | hsa-miR-598-3p |
| hsa-miR-106b-3p | hsa-miR-139-5p | hsa-miR-25-3p | hsa-miR-432-5p | hsa-miR-518e-3p | hsa-miR-654-3p |
| hsa-miR-10a-5p | hsa-miR-149-5p | hsa-miR-25-5p | hsa-miR-433-3p | hsa-miR-518e-5p | hsa-miR-708-5p |
| hsa-miR-10b-5p | hsa-miR-151a-3p | hsa-miR-26a-5p | hsa-miR-4485-3p | hsa-miR-518f-3p | hsa-miR-767-5p |
| hsa-miR-1180-3p | hsa-miR-153-3p | hsa-miR-301a-3p | hsa-miR-454-3p | hsa-miR-519a-3p | hsa-miR-769-5p |
| hsa-miR-124-3p | hsa-miR-17-5p | hsa-miR-301b-3p | hsa-miR-483-3p | hsa-miR-519a-5p | hsa-miR-7706 |
| hsa-miR-1247-5p | hsa-miR-181a-2-3p | hsa-miR-30c-5p | hsa-miR-483-5p | hsa-miR-519b-3p | hsa-miR-874-3p |
| hsa-miR-125a-5p | hsa-miR-181a-3p | hsa-miR-323a-3p | hsa-miR-484 | hsa-miR-519b-5p | hsa-miR-877-5p |
| hsa-miR-125b-1-3p | hsa-miR-181a-5p | hsa-miR-323b-3p | hsa-miR-485-3p | hsa-miR-519c-3p | hsa-miR-887-3p |
| hsa-miR-125b-2-3p | hsa-miR-181b-5p | hsa-miR-324-5p | hsa-miR-485-5p | hsa-miR-519c-5p | hsa-miR-889-3p |
| hsa-miR-125b-5p | hsa-miR-181c-3p | hsa-miR-328-3p | hsa-miR-486-5p | hsa-miR-519d-3p | hsa-miR-891a-5p |
| hsa-miR-1260a | hsa-miR-181c-5p | hsa-miR-331-3p | hsa-miR-487a-3p | hsa-miR-519d-5p | hsa-miR-9-3p |
| hsa-miR-1269a | hsa-miR-181d-5p | hsa-miR-335-3p | hsa-miR-487b-3p | hsa-miR-520a-3p | hsa-miR-9-5p |
| hsa-miR-127-3p | hsa-miR-182-5p | hsa-miR-335-5p | hsa-miR-490-3p | hsa-miR-520a-5p | hsa-miR-92a-1-5p |
| hsa-miR-128-3p | hsa-miR-183-5p | hsa-miR-340-5p | hsa-miR-495-3p | hsa-miR-520c-3p | hsa-miR-92a-3p |
| hsa-miR-1283 | hsa-miR-18a-3p | hsa-miR-342-5p | hsa-miR-498 | hsa-miR-520d-3p | hsa-miR-92b-3p |
| hsa-miR-129-2-3p | hsa-miR-18a-5p | hsa-miR-345-5p | hsa-miR-500a-3p | hsa-miR-520e | hsa-miR-92b-5p |
| hsa-miR-129-5p | hsa-miR-191-5p | hsa-miR-361-5p | hsa-miR-504-5p | hsa-miR-520f-3p | hsa-miR-93-5p |
| hsa-miR-1296-5p | hsa-miR-193b-3p | hsa-miR-3615 | hsa-miR-505-3p | hsa-miR-520g-3p | hsa-miR-941 |
| hsa-miR-1301-3p | hsa-miR-195-5p | hsa-miR-363-3p | hsa-miR-512-3p | hsa-miR-520h | hsa-miR-99a-3p |
| hsa-miR-130a-3p | hsa-miR-197-3p | hsa-miR-370-3p | hsa-miR-515-5p | hsa-miR-522-3p | hsa-miR-99b-3p |
| hsa-miR-130b-3p | hsa-miR-204-5p | hsa-miR-373-3p | hsa-miR-516a-5p | hsa-miR-522-5p | hsa-miR-99b-5p |
| hsa-miR-130b-5p | hsa-miR-20b-5p | hsa-miR-376c-3p | hsa-miR-516b-5p | hsa-miR-523-3p |  |
| hsa-miR-132-3p | hsa-miR-214-3p | hsa-miR-381-3p | hsa-miR-517a-3p | hsa-miR-523-5p |  |
| hsa-miR-1323 | hsa-miR-216a-3p | hsa-miR-382-5p | hsa-miR-517b-3p | hsa-miR-524-3p |  |

**Table S4.** Expression changes of 30 selected miRNAs reported in previous studies.

| miRNA | Previous reports |
| --- | --- |
| hsa-miR-106a-5p | Abu-Elneel *et al*. [2008] (↓) |
| hsa-miR-10a-5p | Ghahramani Seno, M. M. *et al*. [2011] (↑) |
| hsa-miR-128-3p | Abu-Elneel *et al*. [2008] (↑) |
| hsa-miR-129-5p | Abu-Elneel *et al*. [2008] (↑) |
| hsa-miR-130a-3p | Mundalil Vasu *et al*. [2014] (↑) |
| hsa-miR-132-3p | Abu-Elneel *et al*. [2008] (↑); Talebizadeh, Z. *et al*. [2008] (↑); Sarachana *et al*. [2010] (↓) |
| hsa-miR-138-5p | Hirsch, M. M. *et al*. [2018] (↑) |
| hsa-miR-139-5p | Sarachana *et al*. [2010] (↓) |
| hsa-miR-151a-3p | Mundalil Vasu *et al*. [2014] (↓); Hicks, S. D. *et al*. [2020] (↓) |
| hsa-miR-181a-5p | Ghahramani Seno, M. M. *et al*. [2011] (↑); Frye, R. E. *et al*. [2021] (↓) |
| hsa-miR-181b-5p | Ghahramani Seno, M. M. *et al*. [2011] (↑); Mundalil Vasu *et al*. [2014] (↓) |
| hsa-miR-181c-5p | Ghahramani Seno, M. M. *et al*. [2011] (↑) |
| hsa-miR-181d-5p | Abu-Elneel *et al*. [2008] (↓) |
| hsa-miR-183-5p | Kichukova, T. M. *et al*. [2017] (↓) |
| hsa-miR-191-5p | Sarachana *et al*. [2010] (↑); Hicks, S. D. *et al*. [2016] (↑) |
| hsa-miR-193b-3p | Abu-Elneel *et al*. [2008] (↓); Kichukova, T. M. *et al*. [2017] (↓) |
| hsa-miR-195-5p | Sarachana *et al*. [2010] (↑); Mundalil Vasu *et al*. [2014] (↑); Huang, F. *et al*. [2015] (↓) |
| hsa-miR-301a-3p | Kichukova, T. M. *et al*. [2017] (↓) |
| hsa-miR-328-3p | Nakata, M. *et al*. [2019] (↑); Kichukova, T. M. *et al*. [2017] (↓); Nt, P. *et al*. [2018] (↓); Mundalil Vasu *et al*. [2014] (↓) |
| hsa-miR-335-3p | Wu, Y. E. *et al*. [2016] (↑); Hicks, S. D. *et al*. [2016] (↑) |
| hsa-miR-363-3p | Wu, Y. E. *et al*. [2016] (↑); Talebizadeh, Z. *et al*. [2008] (↓) |
| hsa-miR-381-3p | Abu-Elneel *et al*. [2008] (↓) |
| hsa-miR-431-5p | Abu-Elneel *et al*. [2008] (↑) |
| hsa-miR-432-5p | Abu-Elneel *et al*. [2008] (↓) |
| hsa-miR-433-3p | Mundalil Vasu *et al*. [2014] (↓); Jyonouchi, H. *et al*. [2019] (↓) |
| hsa-miR-484 | Abu-Elneel *et al*. [2008] (↑); Wu, Y. E. *et al*. [2016] (↑) |
| hsa-miR-487b-3p | Kichukova, T. M. *et al*. [2017] (↓) |
| hsa-miR-504-5p | Kichukova, T. M. *et al*. [2017] (↓) |
| hsa-miR-574-3p | Huang, F. *et al*. [201] (↓); Jyonouchi, H. *et al*. [2019] (↓) |
| hsa-miR-598-3p | Abu-Elneel *et al*. [2008] (↑) |

**Table S5.** Expression of 30 brain-specific autism-associated miRNAs in male samples.

| miRNA | FC | *p*^a^ | FDR^b^ | Regulation |  | miRNA | FC | *p*^a^ | FDR^b^ | Regulation |
| --- | --- | --- | --- | --- | --- | --- | --- | --- | --- | --- |
| hsa-miR-151a-3p | **2.39** | **8.01E-05** | **1.20E-03** | up |  | hsa-miR-433-3p | 1.57 | 0.17 | 0.32 | up |
| hsa-miR-191-5p | **2.33** | **7.60E-05** | **1.20E-03** | up |  | hsa-miR-132-3p | 1.44 | 0.20 | 0.34 | up |
| hsa-miR-139-5p | **2.00** | **7.47E-04** | **7.47E-03** | up |  | hsa-miR-183-5p | 1.49 | 0.21 | 0.34 | up |
| hsa-miR-181a-5p | 1.67 | **8.28E-03** | **0.04** | up |  | hsa-miR-598-3p | 1.52 | 0.24 | 0.38 | up |
| hsa-miR-195-5p | 1.58 | **7.64E-03** | **0.04** | up |  | hsa-miR-130a-3p | 1.32 | 0.28 | 0.42 | up |
| hsa-miR-432-5p | **2.01** | **6.21E-03** | **0.04** | up |  | hsa-miR-128-3p | 1.79 | 0.33 | 0.48 | up |
| hsa-miR-181b-5p | 1.47 | **0.01** | 0.05 | up |  | hsa-miR-129-5p | 0.90 | 0.39 | 0.50 | down |
| hsa-miR-328-3p | 1.44 | **0.01** | 0.05 | up |  | hsa-miR-181c-5p | 1.62 | 0.40 | 0.50 | up |
| hsa-miR-504-5p | 0.53 | **0.02** | 0.08 | down |  | hsa-miR-335-3p | 1.30 | 0.41 | 0.50 | up |
| hsa-miR-193b-3p | 0.46 | **0.03** | 0.09 | down |  | hsa-miR-381-3p | 0.44 | 0.40 | 0.50 | down |
| hsa-miR-363-3p | 1.33 | **0.03** | 0.09 | up |  | hsa-miR-10a-5p | 1.06 | 0.47 | 0.54 | up |
| hsa-miR-484 | 1.14 | 0.07 | 0.18 | up |  | hsa-miR-431-5p | 0.59 | 0.59 | 0.65 | down |
| hsa-miR-106a-5p | 1.68 | 0.10 | 0.24 | up |  | hsa-miR-574-3p | 1.19 | 0.82 | 0.88 | up |
| hsa-miR-138-5p | 0.54 | 0.17 | 0.32 | down |  | hsa-miR-301a-3p | 1.06 | 0.88 | 0.88 | up |
| hsa-miR-181d-5p | 1.34 | 0.16 | 0.32 | up |  | hsa-miR-487b-3p | 0.91 | 0.87 | 0.88 | down |

Abbreviations: FC, fold change; FDR, false discovery rate.

^a^ Mann–Whitney U test (two-tailed).

^b^ Bold values indicate FDR < 0.05.

**Table S6.** Target genes of 10 significantly differentially expressed miRNAs for ASD candidate risk genes from SFARI database.

| miRNA | Targets of ASD candidate risk genes from SFARI database |
| --- | --- |
| hsa-miR-191-5p | *BRAF*, *NRCAM* |
| hsa-miR-151a-3p | *CECR2*, *NTRK3*, *WNK3* |
| hsa-miR-139-5p | *ANK2*, *ASH1L*, *ASXL3*, *ATP2B2*, *ATRX*, *DDX3X*, *ELAVL2*, *FMR1*, *FOXP2*, *GABBR2*, *HNRNPF*, *HRAS*, *PIK3CA*, *TAOK1* |
| hsa-miR-181a-5p | *ASXL3*, *BAZ2B*, *BIRC6*, *CADPS2*, *CDON*, *CHD7*, *CLASP1*, *CNKSR2*, *CNTNAP2*, *DLG2*, *FAT1*, *FMR1*, *GPD2*, *ITSN1*, *JARID2*, *KANK1*, *KCNJ10*, *KMT2A*, *KMT2C*, *MECP2*, *NAALADL2*, *NBEA*, *NEGR1*, *NFIB*, *NSD2*, *PCDHA5*, *PER2*, *PHF3*, *PTBP2*, *PTEN*, *PTGS2*, *RALGAPB*, *TBL1X*, *TBL1XR1*, *TCF4*, *TNRC6B* |
| hsa-miR-432-5p | *CSDE1*, *DST*, *GNAS*, *LRRC1*, *SETBP1*, *ZBTB20* |
| hsa-miR-181b-5p | *ARNT2*, *BAZ2B*, *BIRC6*, *CADPS2*, *CCNG1*, *CDON*, *CECR2*, *CHD1*, *CPEB4*, *DDX3X*, *DOCK4*, *EGR3*, *EP300*, *FGFR1*, *FMR1*, *FOXP1*, *GABRA4*, *GPD2*, *GRIK2*, *ITSN1*, *JARID2*, *KANK1*, *KDM5A*, *KMT2A*, *KMT2C*, *KMT2E*, *LRBA*, *MAP1A*, *MYO1E*, *NBEA*, *NFIA*, *NFIB*, *NR2F1*, *NRXN1*, *PCDHA2*, *PCDHA4*, *PCDHA5*, *PCDHAC1*, *PCDHAC2*, *PPFIA1*, *PRICKLE2*, *PRKN*, *PTEN*, *RALGAPB*, *RFX3*, *RORB*, *RPS6KA3*, *SCN8A*, *SLC4A10*, *STXBP5*, *TAOK1*, *TBL1XR1*, *TRRAP*, *UNC80*, *WDFY3*, *XRCC6*, *ZBTB20*, *ZMYND11* |
| hsa-miR-195-5p | *AGO1*, *BTAF1*, *BTRC*, *CACNA1E*, *CASK*, *CHD2*, *CSMD1*, *GRM7*, *HECTD4*, *KATNAL1*, *KIF5C*, *LRP2*, *MECP2*, *MIB1*, *MTHFR*, *MYLK*, *MYT1L*, *PCDHA4*, *PCDHA9*, *PHIP*, *PLXNA4*, *PRICKLE2*, *RALGAPB*, *RASSF5*, *RELN*, *RFX3*, *SLC9A6*, *SMURF1*, *SPTBN1*, *SRPRA*, *SRSF11*, *STXBP5*, *SYNJ1*, *TAOK1*, *TBL1XR1*, *TNRC6B*, *ZBTB20* |
| hsa-miR-328-3p | *AGO1*, *HNRNPF*, *MECP2*, *MED13L*, *RALGAPB*, *TCF7L2*, *TSC1* |
| hsa-miR-106a-5p | *AGO1*, *AHNAK*, *ANKRD17*, *ARHGAP5*, *CADM2*, *CSDE1*, *DIP2A*, *EMSY*, *ESR2*, *GPC6*, *INTS6*, *IQSEC2*, *KATNAL1*, *KCNB1*, *KCNJ10*, *KDM6B*, *KMT2A*, *KMT2C*, *KMT5B*, *MECP2*, *MRTFB*, *MTF1*, *MYH9*, *NCKAP5*, *PCDHAC1*, *POLR3A*, *RPS6KA2*, *SCN1A*, *SLC24A2*, *ST8SIA2*, *TBL1XR1*, *TCF4*, *THRA*, *TNRC6B*, *UBE3C*, *UBR5*, *UNC80*, *WAC*, *WDFY3*, *WNK3*, *ZBTB20*, *ZFYVE26*, *ZNF827* |
| hsa-miR-484 | *ADA*, *AGO1*, *AHDC1*, *CAPRIN1*, *CECR2*, *CYFIP1*, *DST*, *EHMT1*, *ELP2*, *EXOC5*, *EXOC6B*, *GIGYF1*, *HDLBP*, *HIVEP2*, *HNRNPH2*, *HNRNPK*, *HOMER1*, *KMT2A*, *KMT2C*, *LDLR*, *MED12L*, *MYH9*, *NEO1*, *NPAS2*, *PER1*, *SRPRA*, *TAOK2*, *TET2*, *TNRC6B*, *TRAF7*, *UBR5*, *WAC* |


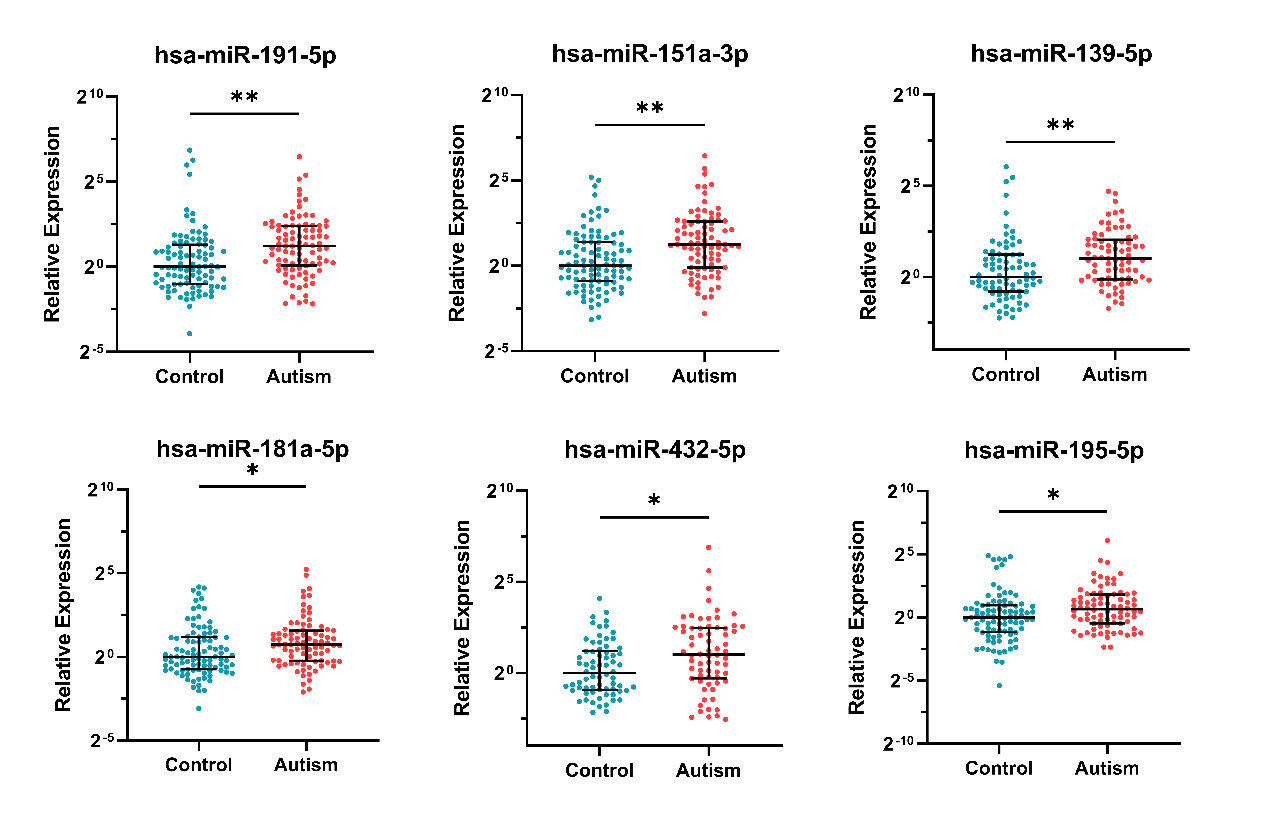
**Figure S1.** Expression of 6 significantly differential expressed miRNAs in male patients with autism and healthy controls.

Mann–Whitney U test was used for statistical analysis and false discovery rate (FDR) was used for multiple testing correction. Data are presented as the median and interquartile range (lines) with each dot representing an individual. * FDR < 0.05, ** FDR < 0.01.
